# Primary Cardiac Lymphoma With Exceptional Long-Term Survival

**DOI:** 10.7759/cureus.100878

**Published:** 2026-01-05

**Authors:** Sohini Ganesuni, Balasubramoniam K R, Rajesh Kannan R, Indu R Nair, Keechilat Pavithran

**Affiliations:** 1 Department of Medical Oncology, Amrita Institute of Medical Sciences and Research Centre, Kochi, IND; 2 Thoracic Surgery, Yashoda Hospitals, Hyderabad, IND; 3 Department of Radiology, Amrita Institute of Medical Sciences and Research Centre, Kochi, IND; 4 Department of Pathology, Amrita Institute of Medical Sciences and Research Centre, Kochi, IND

**Keywords:** cardiac mass, diffuse large b cell lymphoma, long-term survival, primary cardiac lymphoma, r chop

## Abstract

Primary cardiac lymphoma (PCL) is an uncommon extranodal lymphoma confined to the heart or pericardium at diagnosis and can mimic other cardiac disorders because of its nonspecific presentation. We report a 53-year-old man diagnosed in 2015 with diffuse large B-cell lymphoma involving the right atrium. He underwent surgical excision followed by six cycles of R-CHOP chemoimmunotherapy. Serial PET/CT and echocardiography confirmed sustained complete metabolic remission, and he remains disease-free 128 months after treatment. This case highlights the potential for prolonged survival in PCL when timely diagnosis, accurate histopathological confirmation, and appropriate therapy are provided.

## Introduction

Primary cardiac lymphoma (PCL) is an exceedingly rare form of extranodal lymphoma that involves the heart or pericardium without evidence of systemic disease at the time of diagnosis [1]. Primary cardiac tumors themselves are uncommon, with an estimated incidence of 0.001-0.03% in autopsy series. Among these, approximately 75-80% are benign, most commonly atrial myxomas, while 20-25% are malignant, with sarcomas accounting for the majority of malignant cases [2,3]. Lymphomas constitute only a small fraction of malignant cardiac tumors.

PCL represents less than 2% of all primary cardiac tumors and a very small proportion of extranodal non-Hodgkin lymphomas [4,5]. It occurs more frequently in immunocompromised individuals, particularly those with HIV infection or post-transplant status, and typically presents in late adulthood with a slight male predominance. Clinical manifestations are often non-specific and depend on tumor size and location, including chest pain, heart failure, arrhythmias, pericardial effusion, or constitutional symptoms, which frequently delay diagnosis.

Multiple imaging modalities, including transthoracic and transesophageal echocardiography, cardiac computed tomography, magnetic resonance imaging, and positron emission tomography, are often required to evaluate cardiac involvement and disease extent. However, definitive diagnosis relies on histopathological and immunohistochemical confirmation [6]. Given its rarity and non-specific presentation, PCL is often misdiagnosed as other benign or malignant cardiac tumors, underscoring the importance of heightened clinical suspicion and timely intervention.

## Case presentation

A 53-year-old man presented in February 2015 with intermittent fever of 2-3 months’ duration and a recent onset of back pain, abdominal bloating, and retrosternal discomfort. He did not report classical B symptoms such as night sweats, unintentional weight loss, or pruritus; however, he noted reduced appetite. The Karnofsky Performance Status score at presentation was 90, indicating good functional capacity. His general condition was stable, and abdominal ultrasonography was unremarkable. A 2-dimensional echocardiogram (2D ECHO) revealed an irregularly shaped mass in the right atrium, occupying > 80% of the right atrial cavity and measuring 5.6 x 3.9 cm. The inferior vena cava (IVC) measured 1.2 cm with normal respiratory variation, and there was no mass extending into the IVC. The left atrium was free of mass. Further evaluation with TEE revealed a large right atrial mass measuring 6.4 x 4.7 cm. The mass was attached to the interatrial septum, causing bulging into the left atrium, with an extension of 5 mm into the superior vena cava (SVC) with mediastinal lymphadenopathy. Computed tomography (MDCT) of the chest and abdomen revealed a large atrial mass with mediastinal lymph nodes and lung nodules.

**Figure 1:**
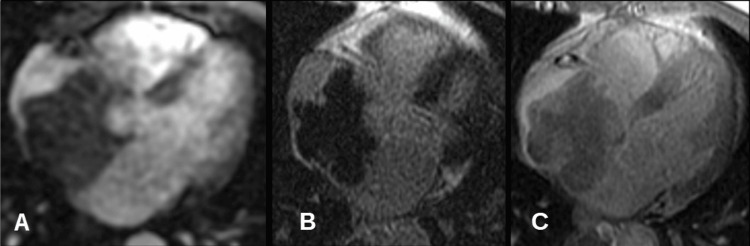
Post-contrast images. On the rest perfusion image (A), the mass showed minimal enhancement. Post contrast late gadolinium sequence with inversion time of 230 ms (B) showed no enhancement of the mass and was not suppressed with inversion time of 600 ms (C).

**Figure 2:**
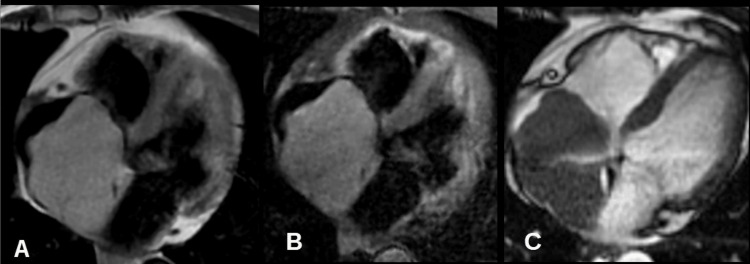
Axial image of the heart. Large right atrial mass appears iso to hyperintense to myocardium on double IR (A) and triple IR (B) sequences and isointense on SSFP (C) images

Cardiac MRI identified a well-circumscribed lesion within the right atrium measuring 73 × 52 mm, with superior extension into the superior vena cava and inferior contact with the suprahepatic inferior vena cava. The mass demonstrated a broad attachment to the interatrial septum (Figure 1), which highlights the iso- to hyperintense appearance of the lesion on double- and triple-inversion recovery sequences. (Figure 2) Further demonstrates the absence of significant contrast enhancement on late gadolinium imaging. The provisional diagnosis was cardiac myxoma.

**Figure 3:**
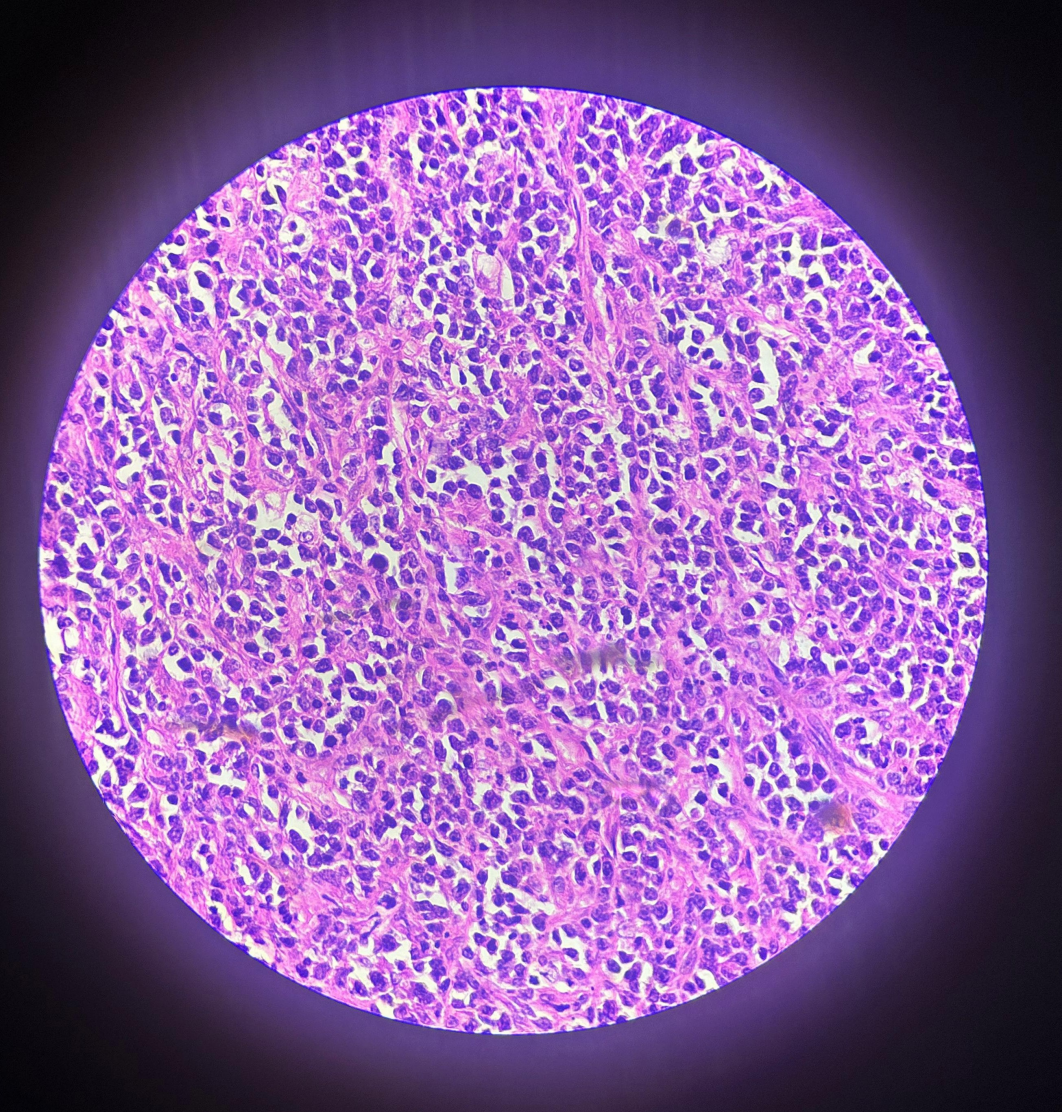
Histopathology (H&E stain, ×40) showing sheets of medium-sized atypical lymphoid cells with irregular nuclei, coarse chromatin, and brisk mitotic and apoptotic activity. Tingible body macrophages, which are macrophages containing phagocytosed apoptotic cellular debris, create a focal starry-sky pattern. Scattered plasma cells, small lymphocytes, and occasional eosinophils are also identified.

The patient underwent complete removal of both interatrial septa, implantation of a bovine pericardial patch between the atria, tricuspid valve annuloplasty, and atrial septal defect (ASD) closure in March 2015. The surgical histopathology report showed a poorly differentiated neoplasm. On immunohistochemistry (IHC), cells were positive for LCA, CD20, PAX-5, BCL2, MUM-1 and negative for CD3, CD10, Cyclin D1, CD5, CD23, BCL6, CD138 and TdT(terminal deoxynucleotidyl transferase). Ki-67 was 80%, and the final diagnosis was non-Hodgkin’s lymphoma of the diffuse large B-cell lymphoma subtype (Figure 3).

**Figure 4:**
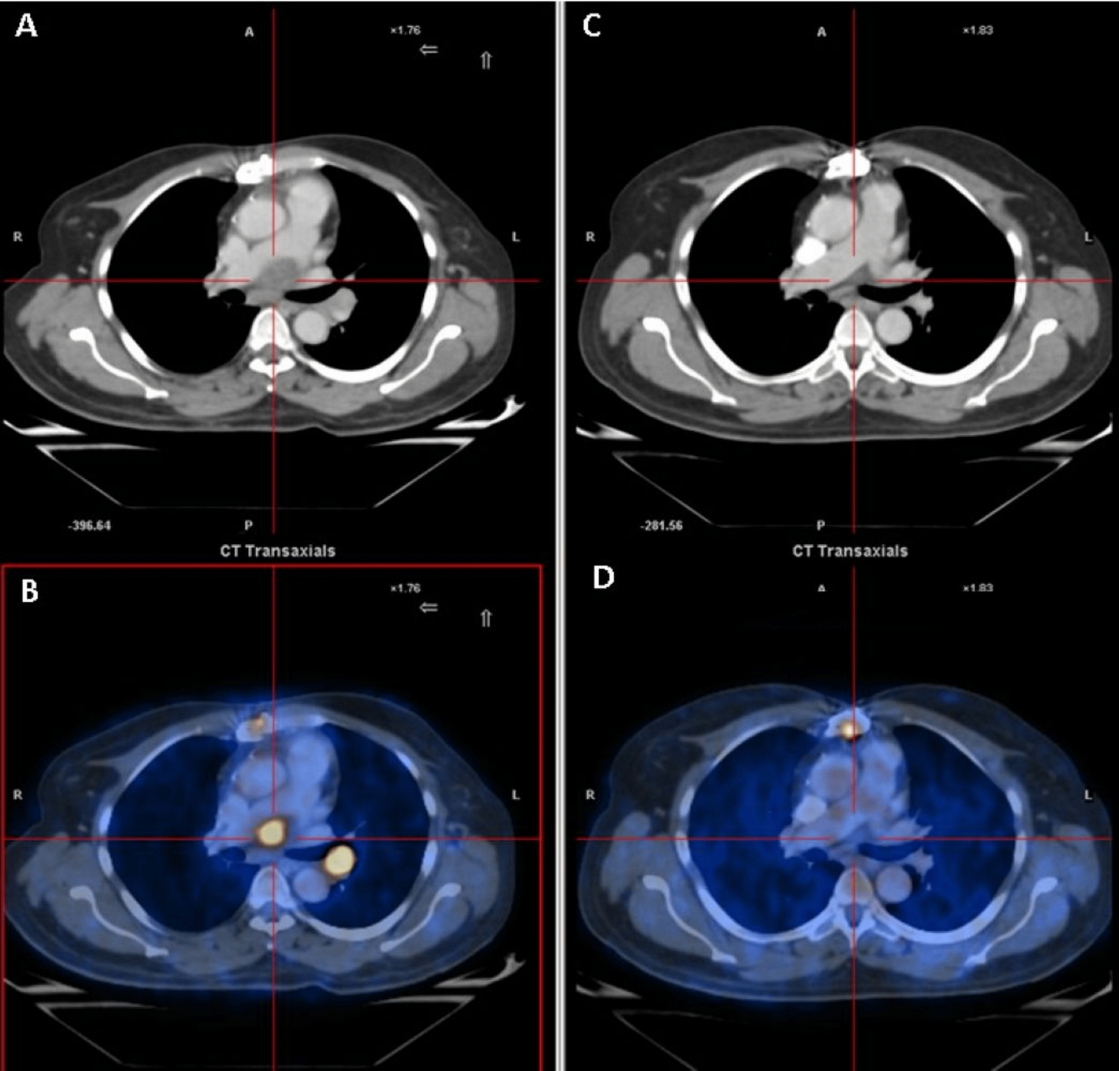
Comparison of PET-CT imaging before and after chemotherapy. Axial CT image from the postoperative PET-CT showing soft-tissue thickening along the interatrial septum (A). Corresponding fused PET-CT image demonstrating intense FDG uptake at the same site (B). Axial CT image after four cycles of R-CHOP chemotherapy showing marked reduction of the lesion (C). Corresponding fused PET-CT image demonstrating near-complete resolution of metabolic activity (D).

Postoperatively, positron emission tomography (PET) revealed multiple FDG-avid conglomerate discrete mediastinal, left hilar, and left para-oesophageal lymph nodes. Additionally, an FDG-avid ill-defined soft tissue lesion was noted along the interatrial septum, extending into the right atrium. He was started on the rituximab-cyclophosphamide, vincristine, adriamycin, and prednisolone (R-CHOP) regimen, and interim whole-body 18FDG PET/CT after four cycles showed regression of the lesions with complete metabolic response (Figure 4). He completed six cycles of R-CHOP by August 2015. Treatment was well tolerated, with no dose reductions or treatment delays, and routine laboratory monitoring during chemotherapy revealed no clinically significant hematologic or biochemical toxicities. The patient achieved remission, which was sustained during subsequent follow-up visits

The most recent PET/CT scan, performed in August 2024, demonstrated a complete metabolic response. Transthoracic echocardiography in March 2025 revealed dilation of the left atrium (LA), right atrium (RA), and right ventricle (RV) with preserved left ventricular (LV) systolic function. No residual mass was identified in the RA, and no abnormal flow was observed across the interatrial septum (IAS). The patient remained asymptomatic and continued to perform well. He exhibited an exceptionally good response, achieved a disease-free survival of 128 months, and was still in remission, with no significant adverse effects.

## Discussion

PCL is frequently diagnosed late or even post‑mortem because of its non-specific symptoms and the difficulty of accessing intracardiac tissue for diagnosis. Secondary cardiac lymphoma (SCL) involvement by lymphoma is more common but often clinically silent and carries a poor prognosis [7]. PCLs tend to arise in the right heart, particularly near the atrioventricular groove, likely due to lymphatic drainage patterns that channel lymphatic return toward the right atrium [8,9]. Outcomes for PCL vary widely and depend on histologic subtype, disease extent, and treatment strategy.

When reviewing our case of primary cardiac lymphoma, it is essential to consider the clinical presentation, diagnostic modalities, and treatment strategies. Our patient presented with nonspecific symptoms that were frequently attributed to other systemic causes. Advanced imaging modalities, including TEE and cardiac MRI (CMR), play a crucial role in revealing the extent of the mass and its involvement with adjacent structures, facilitating a more accurate diagnosis. Although preoperative imaging suggested cardiac myxoma, a preoperative biopsy was not performed because of the initial suspicion of a benign cardiac tumour. Complete surgical excision confirmed the diagnosis of diffuse large B-cell lymphoma.

Increased age has been associated with worse survival outcomes in patients with primary cardiac lymphoma [9]. Survival outcomes are better among those treated with chemotherapy, and the addition of rituximab has been shown to further improve prognosis in a statistically significant manner [10].

In this patient, long-term remission was likely supported by several favorable factors, including localized disease at presentation, timely administration of R-CHOP therapy, and the absence of major comorbidities or immunosuppression. Although Sultan et al. [10] reported a median survival of 45.4 months with an interquartile range of 5.6-120.4 months, long-term survival exceeding 10 years remains rare. This case exceeds that expectation, demonstrating durable remission and excellent functional recovery.

Although malignant lymphomas are generally radiosensitive and radiotherapy is commonly used in selected settings, its role in primary cardiac lymphoma is limited due to the risk of long-term cardiac toxicity, including cardiomyopathy, coronary artery disease, valvular dysfunction, and conduction abnormalities. Consequently, systemic chemoimmunotherapy remains the preferred treatment approach for cardiac involvement.

In recent years, advanced therapeutic options for aggressive B-cell lymphomas, including CD19-directed CAR T-cell therapies and CD20×CD3 bispecific antibodies, have demonstrated efficacy in relapsed or refractory settings. However, these approaches are typically reserved for later lines of therapy and have not been specifically studied in primary cardiac lymphoma.

## Conclusions

Primary cardiac lymphoma (PCL) is challenging to diagnose owing to its insidious onset and non-specific clinical manifestations. Advanced imaging modalities, particularly transesophageal echocardiography (TEE) and cardiac magnetic resonance imaging (CMR), are essential for diagnosing and assessing cardiac and extracardiac involvement. Diagnosis is often delayed because patients may remain asymptomatic for long periods of time, which contributes to less favorable clinical outcomes. Chemotherapy is the cornerstone of treatment. The addition of immunotherapy (e.g., rituximab) has significantly improved outcomes. This case highlights that early recognition, accurate histopathological diagnosis, and timely initiation of chemoimmunotherapy can result in sustained complete remission and long-term disease-free survival even in this rare and aggressive malignancy.
